# Clinical Outcome of Ultrasound-Detected Perforated Necrotizing Enterocolitis without Radiographic Pneumoperitoneum in Very Preterm Infants

**DOI:** 10.3390/jcm12051805

**Published:** 2023-02-23

**Authors:** Myoung Kyoung Kim, Tae Yeon Jeon, Kyunga Kim, Yu Jin Kim, So-Young Yoo, Ji Hye Kim, Yun Sil Chang, Sanghoon Lee, Jeong-Meen Seo, Sung-Hoon Moon

**Affiliations:** 1 Department of Radiology and Center for Imaging Science, Samsung Medical Center, Sungkyunkwan University School of Medicine, Seoul 06351, Republic of Korea; 2 Biomedical Statistics Center, Research Institute for Future Medicine, Samsung Medical Center, Sungkyunkwan University School of Medicine, Seoul 06351, Republic of Korea; 3 Department of Surgery, Samsung Medical Center, Sungkyunkwan University School of Medicine, Seoul 06351, Republic of Korea; 4 Department of Pediatrics, Samsung Medical Center, Sungkyunkwan University School of Medicine, Seoul 06351, Republic of Korea; 5 Department of Internal Medicine, Hallym University Sacred Heart Hospital, Anyang 14068, Republic of Korea

**Keywords:** enterocolitis, necrotizing, infant, extremely premature, ultrasonography, pneumoperitoneum

## Abstract

Objectives: To investigate the clinical outcomes of ultrasound (US)-detected perforated necrotizing enterocolitis (NEC) without radiographic pneumoperitoneum in very preterm infants. Methods: In this single-center retrospective study, very preterm infants who underwent a laparotomy for perforated NEC during their neonatal intensive care unit stay were classified into two groups according to the absence or presence of pneumoperitoneum on radiographs (the case versus the control groups). The primary outcome was death before discharge, and the secondary outcomes included major morbidities and body weight at 36 weeks postmenstrual age (PMA). Results: Of the 57 infants with perforated NEC, 12 (21%) had no pneumoperitoneum on the radiographs and were diagnosed with perforated NEC on the US. In the multivariable analyses, the primary outcome of death before discharge was significantly lower in infants with perforated NEC without radiographic pneumoperitoneum than in those with perforated NEC and radiographic pneumoperitoneum (8% [1/12] vs. 44% [20/45]; adjusted odds ratio [OR], 0.02; 95% confidence interval [CI], 0.00–0.61; *p* = 0.025). The secondary outcomes (short bowel syndrome, total parenteral nutrition dependence for 3 months or more, the length of their hospital stay, a bowel stricture requiring surgery, sepsis after the laparotomy, acute kidney injury after the laparotomy, and body weight at 36 weeks PMA) did not differ significantly between the two groups. Conclusions: Very preterm infants with US-detected perforated NEC without radiographic pneumoperitoneum had a lower risk of death before discharge than those with perforated NEC and radiographic pneumoperitoneum. Bowel USs may have a potential role in surgical decision-making in infants with advanced NEC.

## 1. Introduction

Necrotizing enterocolitis (NEC) is one of the most common and devastating bowel diseases in infants, especially in those born prematurely [1]. Despite the ever-improving survival rate of very preterm infants, the number of deaths attributed to NEC has increased [2]. The overall mortality rate for infants with NEC ranges between 20% and 30%, with a rate as high as 50% for very low birth weight infants requiring surgery [3,4]. The higher mortality rate following bowel perforation suggests that early detection and prompt management could potentially improve the prognosis of NEC. Therefore, imaging may play an important role in the management of infants with NEC.

The only absolute indication for laparotomy in NEC is evidence of pneumoperitoneum on radiographs [4]. However, diagnosing bowel perforation is not straightforward because perforation can occur without radiographic evidence of pneumoperitoneum [5,6]. The use of ultrasound (US) as an imaging adjunct to radiography in NEC has increased in recent years [1,7,8]. Previous US studies have shown that complex ascites, focal fluid collections, and the absence of bowel perfusion are reliable US signs of bowel perforation, even in the absence of pneumoperitoneum on radiographs [9].

Appropriate surgical decision-making has been highlighted as an area with the potential to improve outcomes. However, indications for laparotomy in NEC in the absence of pneumoperitoneum on radiographs have been a subject of ongoing debate in clinical practice. One unclear aspect is whether the need for and the timing of the laparotomy influence the prognosis of infants with NEC who are suspected of having bowel perforation on US but show no pneumoperitoneum on radiographs. Few studies have investigated this potential association.

The aim of the present study was to investigate the clinical outcomes of US-detected perforated NEC without radiographic pneumoperitoneum in very preterm infants.

## 2. Materials and Methods

### 2.1. Study Population

This retrospective study was conducted according to the guidelines of the Declaration of Helsinki and was approved by the Institutional Review Board (IRB) of the Samsung Medical Center (2020-12-131). The IRB also waived the need to obtain informed consent.

A retrospective search of data from January 2010 to August 2020 in the electronic database of the Department of Surgery and Neonatology at our institution identified all infants who met the following criteria: (a) infants born prematurely before 32 completed weeks of gestation and admitted to the neonatal intensive care units (NICUs) of our tertiary care center (*n* = 1466) and (b) infants who underwent laparotomy for bowel perforation during their NICU stay (*n* = 95). Of the 95 infants with bowel perforation, 38 were excluded for the following reasons: (a) 28 infants who underwent laparotomy for reasons other than perforated NEC (spontaneous bowel perforation, *n* = 8; meconium plug syndrome, *n* = 10; meconium peritonitis, *n* = 3; ileal or jejunal atresia, *n* = 2; midgut volvulus, *n* = 2; bowel perforation during enema examination, *n* = 2; and incarcerated inguinal hernia with bowel perforation, *n* = 1); and (b) 10 infants who were referred from an outside hospital for surgery. None of the infants had any congenital anomalies, metabolic diseases, or coagulation disorders. Perforated NEC was evaluated using abdominal radiographs and/or US examinations and confirmed intraoperatively or histopathologically. The intraoperative diagnosis of a perforated intestine in a newborn with NEC was defined as (1) apparent fecal material in the peritoneal cavity with visible perforation along the intestinal wall or (2) abscess cavity with or without fecal material, suggesting walled-off perforation. Finally, 57 very preterm infants (male to female ratio, 33:24; median gestational age at birth, 24.6 weeks, interquartile range (IQR) 23.4–25.4; median birth weight, 620 g, IQR 540–730) were included in this study.

Of these 57 infants with intraoperatively or histopathologically confirmed perforated NEC, the case group (*n* = 12) consisted of infants who had no pneumoperitoneum on preoperative abdominal radiographs and who were diagnosed with perforated NEC using US. The control group (*n* = 48) comprised infants diagnosed with perforated NEC who displayed pneumoperitoneum on radiographs.

### 2.2. Clinical Data Collection and Outcome Measures

The infants’ demographic data included gestational age at birth, birth weight, sex, Apgar scores determined at 1 and 5 min, small for gestational age, and postnatal age at laparotomy. Maternal clinical data were also collected, including the detection of chorioamnionitis, premature rupture of the membrane, and antenatal corticosteroid use. If present, the following conditions were documented before laparotomy: high-grade intraventricular hemorrhage (IVH, a grade ≥III of the Papile classification), neonatal sepsis, acute kidney injury (AKI), nonsteroidal anti-inflammatory drug (NSAID) use, and high-frequency oscillatory ventilation (HFOV) support. Preoperative platelet counts were also documented. Neonatal sepsis was defined as culture-proven sepsis or definite clinical signs of sepsis with a negative culture [10].

The primary outcome was death before discharge from the NICU. The secondary outcomes included short bowel syndrome, total parenteral nutrition dependence for 3 months or more, length of hospital stay, bowel stricture requiring surgery, sepsis after laparotomy, AKI after laparotomy, and body weight at 36 weeks postmenstrual age (PMA).

### 2.3. Image Acquisition and Analysis

Regarding our institutional imaging protocol for NEC, the initial strategy for infants with suspected NEC includes a combination of radiographs followed by a bowel US. In infants with confirmed NEC, the timing of follow-up radiographs usually varies from 6 to 24 h, and it depends on the severity of the NEC. Subsequent US is selectively used per clinical discretion, and it is indicated when indeterminate radiographs, suspected perforation not evident radiographically, clinical deterioration, and radiographic findings unrelated to clinical presentation [11].

Before laparotomy, all infants underwent abdominal radiography, including supine anteroposterior (*n* = 57) and lateral shoot-through (*n* = 55) examinations. Radiographs were obtained using portable X-ray equipment at 45–60 kVp and 1–2 mA. Two pediatric radiologists, with 19 and 12 years of post-fellowship experience, evaluated each radiograph by consensus and were blinded to the clinical and US findings. Radiographs were evaluated for bowel gas patterns and the presence or absence of pneumatosis, portal venous gas, and pneumoperitoneum. The bowel gas pattern was categorized into four types: (1) normal bowel gas, (2) decreased bowel gas or gasless, (3) ileus without elongated loops, and (4) ileus with elongated loops [12].

Of the 57 infants with perforated NEC, 48 underwent bowel US examinations conducted by one of three pediatric radiologists (J.H.K., S.Y.Y., or T.Y.J., with 13, 9, and 4 years of experience in pediatric US, respectively, at the time when they each first evaluated the study subjects). In our NICU, US examinations were performed on infants with clinically suspected NEC using the technique described by Faingold et al. [9]. The US system consisted of a LOGIQ E9 (GE Healthcare) with high-frequency linear-array transducers (9 MHz or 6–15 MHz). Fasting was not required before the US examination, and no infants were sedated. At one-month intervals after the abdominal radiographic analysis, the same readers evaluated each US examination by consensus while blinded to the clinical and radiographic findings. The last US examination was performed within 1 week before laparotomy was analyzed.

According to the guidelines by Faingold et al. [9], the bowel US was evaluated for the presence or absence of the following abnormalities: (1) pneumoperitoneum, (2) portal venous gas, (3) pneumatosis, (4) abdominal fluid (complex ascites, focal fluid collections, and simple ascites), (5) bowel wall thickening, and (6) absent or decreased bowel perfusion on color Doppler US. Abdominal fluid was considered complex when echogenic materials or septations were noted. US findings indicative of bowel perforation were defined as the presence of complex ascites, focal fluid collections, or free gas [9,12,13,14,15,16,17].

### 2.4. Statistical Analysis

All statistical analyses were performed using SAS version 9.4 software (SAS Institute) and Rex version 3.6.0 (RexSoft Inc., Seoul, Republic of Korea). Statistical significance was set at *p* < 0.05. Baseline characteristics, imaging findings, and outcomes were compared between infants with perforated NEC without radiographic pneumoperitoneum and those with perforated NEC and radiographic pneumoperitoneum. The χ^2^ or Fisher’s exact test was used for categorical variables, and the Wilcoxon rank sum test or Student’s *t*-test was used for nonparametric continuous variables. The primary and secondary outcome measures were compared between the two groups using bivariate analysis. The adjusted odds ratios (ORs) and 95% confidence intervals (CIs) were calculated by binary logistic regression or linear regression analysis for primary and secondary outcomes in infants with perforated NEC with or without radiographic pneumoperitoneum. Gestational age, HFOV support, preoperative platelet count, and the interval between the first detection of complex ascites or focal fluid collection on US and laparotomy were used as confounding variables in the multivariable analyses.

## 3. Results

### 3.1. Demographics and Clinical Characteristics

The demographic data and clinical characteristics of each study group are presented in Table 1. No significant differences were noted in gestational age, birth weight, sex, Apgar scores, small for gestational age, maternal clinical data, high-grade IVH, AKI, NSAID use, HFOV support, or preoperative platelet counts between the two groups. Neonatal sepsis occurred less commonly in infants with US-detected perforated NEC without radiographic pneumoperitoneum (67%, 8/12) than in infants with perforated NEC and radiographic pneumoperitoneum (98%, 44/45) (*p* = 0.006). At the laparotomy, the case group (median 30 days; IQR 22–45.5) was older than the control group (median 20 days; IQR 12–31) (*p* = 0.018).

### 3.2. Imaging Findings

The imaging findings of abdominal radiographs and US were compared between the two groups (Table 2).

#### 3.2.1. Abdominal Radiographs

The bowel gas pattern, presence of pneumatosis, and presence of portal venous gas on the abdominal radiographs did not differ between the two groups. Ileus with elongated loops was common in both groups (75% [9/12] vs. 69% [31/45]; *p* = 1.000).

#### 3.2.2. Temporal Relation between Abdominal Radiographs and Bowel US

**Figure 1:**
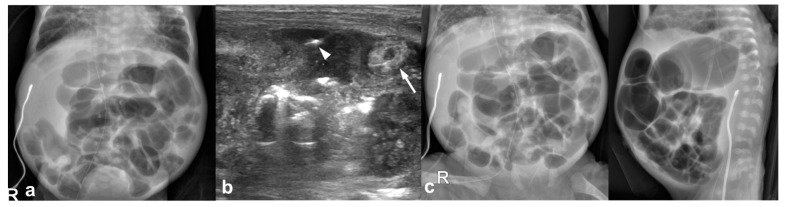
22-day-old girl (born at gestational age of 25 weeks and 3 days) with US-detected perforated necrotizing enterocolitis without radiographic pneumoperitoneum; this infant survived. (**a**) An abdominal radiograph obtained 7 h before US shows ileus with elongated loops and no pneumoperitoneum. (**b**) Preoperative US images show bowel wall thickening (arrow) and free air within the complicated ascites (arrowhead). (**c**) Preoperative abdominal radiographs obtained 30 min after US show no pneumoperitoneum.

**Figure 2:**
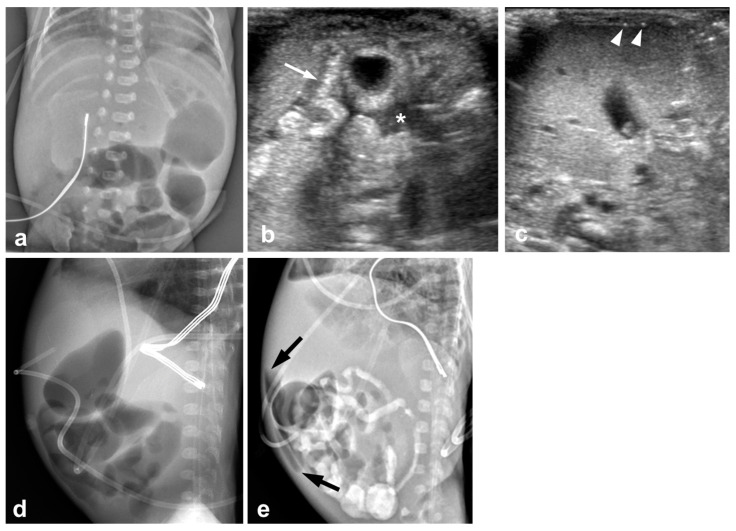
12-day-old boy (born at gestational age of 24 weeks and 1 day) with perforated necrotizing enterocolitis and radiographic pneumoperitoneum; this infant died 11 days after laparotomy. (**a**) An abdominal radiograph obtained 1 day before US shows nonspecific distension of the bowels without free air. (**b**,**c**) Preoperative US images show increased intestinal wall echogenicity and wall thickening (arrow), complex ascites (asterisk), and free air (arrowheads) between the hepatic surface and the anterior abdominal wall. (**d**) Preoperative abdominal radiographs obtained 1 h after US show no pneumoperitoneum. (**e**) Pneumoperitoneum (arrows) appears on abdominal radiographs obtained 35 h after US.

**Figure 3:**
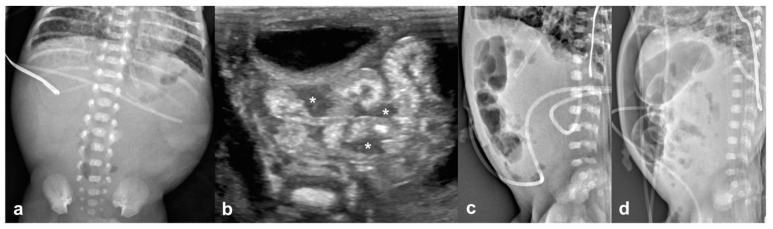
21-day-old boy (born at gestational age of 23 weeks and 2 days) with perforated necrotizing enterocolitis and radiographic pneumoperitoneum; this infant died 38 days after laparotomy. (**a**) An abdominal radiograph obtained 1 day before US shows a relatively gasless abdomen without pneumoperitoneum. (**b**) Preoperative US images show increased intestinal wall echogenicity and wall thickening, and complex ascites (asterisks). (**c**) Preoperative abdominal radiograph obtained 4 h after US showed no free air. (**d**) Pneumoperitoneum appears on abdominal radiographs obtained 4 days after US.

One infant in the case group showed small amounts of free air on the US, but no pneumoperitoneum was observed on the radiographs performed immediately after the US study (Figure 1). Of the 36 controls with radiographic pneumoperitoneum who underwent US, free air was found during the US in only eight infants (22%). Of these eight control infants showing free air on the US, two had no pneumoperitoneum on the radiographs at the time of the US but later presented pneumoperitoneum on the radiographs at 24 and 35 h (Figure 2). All infants in the case group had abdominal fluid (complex ascites, *n* = 11; focal fluid collections, *n* = 4), while 76% (29/36) of the infants in the control group had abdominal fluid (complex ascites, *n* = 26; focal fluid collections, *n* = 6; simple ascites, *n* = 2). Complex ascites or focal fluid collections were commonly observed in both groups (100% [12/12] vs. 75% [27/36]; *p* < 0.001). Pneumoperitoneum on the radiographs appeared at an average of 4.4 ± 5.2 days (range, 0–27 days) after complex ascites or focal fluid collections were first detected on the US in 27 patients of the control group (Figure 3). The interval between preoperative US and laparotomy was shorter in the case group than in the control group (2.2 ± 2.6 days vs. 3.2 ± 2.3 days; *p* = 0.029). The mean interval between the first detection of complex ascites or focal fluid collections on the US and laparotomy showed a tendency of a shorter period in the case group than in the control group (4.3 ± 4.1 days vs. 7.1 ± 6.0 days), but there was no statistical significance (*p* = 0.158).

### 3.3. Clinical Outcomes

Only one infant died in the case group (8%); the period from the first detection of complex ascites on the US to laparotomy in this case was 14 days, the longest in the case group. By contrast, 44% of the infants in the control group died before discharge. Consequently, death before discharge (the primary outcome) was less prevalent in the case group than in the control group (8% [1/12] vs. 44% [20/45]; *p* = 0.040) in the bivariate analysis. The secondary outcome of AKI after laparotomy was also less prevalent in the case group than in the control group (8% [1/12] vs. 47% [21/45]; *p* = 0.019). The body weight at 36 weeks PMA was higher in the case group than in the control group (1690 vs. 1535 g; *p* = 0.007) (Table 3).

Multivariable analyses revealed that the primary outcome of death before discharge was significantly lower in infants with perforated NEC without radiographic pneumoperitoneum than in those with perforated NEC and radiographic pneumoperitoneum (adjusted OR, 0.02; 95% CI, 0.00–0.61; *p* = 0.025). The secondary outcomes (short bowel syndrome, total parenteral nutrition dependence for 3 months or more, length of hospital stay, bowel stricture requiring surgery, sepsis after laparotomy, acute kidney injury after laparotomy, and body weight at 36 weeks PMA) did not differ significantly between the two groups (Table 3).

## 4. Discussion

Our study demonstrated that very preterm infants with US-detected perforated NEC without radiographic pneumoperitoneum showed a lower risk of death before discharge compared to those with perforated NEC and radiographic pneumoperitoneum. All 12 infants, with no radiographic evidence of free air but with US findings of perforation, were confirmed as having perforation intraoperatively or histopathologically. The radiographic pneumoperitoneum appeared an average of 4.4 days after the US finding of complex ascites or focal fluid collections, suggesting that US may be useful for detecting the early stage of perforated NEC prior to evidence of radiographic pneumoperitoneum. Therefore, our study confirmed the critical role of US in guiding clinical decision-making in very preterm infants with NEC.

NEC is an ischemic condition in the bowel mucosa that leads to inflammation and necrosis. As NEC progresses, mucosal injury and full-thickness bowel necrosis occur, leading to thinning of the bowel wall and, eventually, perforation [18,19].

Abdominal radiography is the standard modality for infants with clinically suspected NEC or for identifying pneumoperitoneum. However, radiographs alone cannot be used to evaluate NEC because of their lack of sensitivity and specificity [5,6,20,21]. Pneumoperitoneum can often be missed on radiographs, and not all cases of bowel perforation show pneumoperitoneum on radiographs [6,12,13,14,15,16,17]. Munaco et al. [6] reported that only 55% of patients with perforated NEC had radiographic evidence of pneumoperitoneum. In addition, the use of radiography for the diagnosis and staging of NEC is limited in most immature infants. The typical radiographic hallmarks of NEC, such as pneumatosis (29% vs. 100%, *p* < 0.001) and portal venous gas (10% vs. 47%, *p* < 0.01), were less frequently detected in very preterm infants than in term infants in a previous study [21].

The use of US in NEC has been described since the 1980s as a complementary imaging modality to radiography [22], and its routine use in NEC has steadily increased in recent years [1,7,8]. A recent systematic review and meta-analysis revealed that several US features, including complex ascites, focal fluid collections, absent peristalsis, and pneumoperitoneum, were associated with surgery or patient demise [23]. The major advantage of US over radiography is its ability to assess the characteristics of the abdominal fluid (simple ascites, complex ascites, or focal fluid collections). The present results demonstrated that complex ascites and focal fluid collections associated with bowel perforation occurred in the absence of pneumoperitoneum on radiographs, in agreement with the findings of other studies [9,12,13,14,15,16,17]. Moreover, two infants in the present study showed free air only on the US but later showed pneumoperitoneum on the radiographs. This led us to infer the following: (1) complex ascites or focal fluid collections alone can be seen prior to the appearance of pneumoperitoneum on radiographs or US in the early stage of perforated NEC, and (2) pneumoperitoneum on radiographs may indicate a late stage of perforated NEC. US is also superior to radiography for the direct visualization of bowel perfusion [9,15,16,17,24]. Faingold et al. [9], in 2005, were the first to document the utility of Doppler US for assessing bowel necrosis in 30 neonates with NEC, and they found that a lack of bowel perfusion was a sign of clinical deterioration. However, Faingold [25] recently noted that not all cases of decreased bowel perfusion indicate a poor prognosis; in some cases, bowel perfusion and the clinical course improved.

The optimal timing and follow-up frequency of bowel USs have not been well defined for determining the need for surgery in infants with NEC. The present findings indicate that a bowel US is most helpful when used in infants who have ileus with elongated loops on radiographs or in infants with NEC at modified Bell stage II or higher. Similar to our results, Silva et al. [12] reported that the detection of gas-filled intestinal dilatation with elongated loops on radiographs might be related to bowel necrosis and sealed perforation in the clinical setting of NEC. We also suggest a short-term follow-up US within 24 h if the bowel US is uncertain in infants with advanced NEC without radiographic pneumoperitoneum.

The limitations of this study include its retrospective design and the relatively small number of subjects who had US-detected perforated NEC without radiographic pneumoperitoneum. In addition, not all patients with NEC might follow the same imaging protocol. Nonetheless, the inclusion of very preterm infants and the single-center nature of this study may mean that less variation in clinical treatment protocols might be a strength of this study. Other limitations include the lack of consensus on indications for surgery at our institution. The decision and timing of surgery in preterm infants with NEC often require careful judgment and balancing of the benefits and risks through a multidisciplinary team approach. Surgery in a critically ill infant may help preserve the viable bowel, but a clinically unstable infant may not be able to withstand the burden of surgery. One substantial challenge in clinical practice is identifying infants requiring NEC surgery when no pneumoperitoneum is evident on radiographs. A desirable solution would be to establish an objective evaluation system that includes clinical, laboratory, and radiographic findings to determine the need for surgery for infants with NEC in the absence of pneumoperitoneum. In the present study, a bowel US was used selectively based on clinical discretion rather than as part of a routine evaluation for NEC. The neonatal surgical teams usually do not request a bowel US in situations where clear surgical decisions can be made based on clinical and radiographic findings. US was requested only when the surgical indications were questionable in infants with advanced NEC. Our study results suggested the complementary nature of needing both radiographs and US for infants with NEC, as 25% (9/36) of the infants with radiographic pneumoperitoneum did not show the US findings indicative of perforated NEC. Both abdominal radiographs and US may be needed to diagnose perforated NEC. In addition, US findings alone may not be used as an exclusive diagnostic tool for perforated NEC in clinical practice. The surgical decision-making in advanced NEC needs a combination of imaging findings and clinical features (e.g., clinical deterioration despite maximal medical therapy). A final limitation is that we could not establish the criteria to differentiate perforated and unperforated NEC. Follow-up studies are needed to create the criteria to diagnose perforated NEC based on various radiographic and US features.

In conclusion, very preterm infants who had US-detected perforated NEC without radiographic pneumoperitoneum showed a lower risk of death before discharge than those who had perforated NEC and radiographic pneumoperitoneum. This study reaffirmed that complex ascites and focal fluid collections indicated bowel perforation, even in the absence of pneumoperitoneum on the radiographs. In addition, pneumoperitoneum might be a late finding of bowel perforation. Bowel USs may be essential for surgical decision-making in infants with advanced NEC without radiographic pneumoperitoneum, and it could potentially change the clinical outcomes of infants with NEC.

## Figures and Tables

**Table 1:** Demographics and clinical characteristics for study population.

Variables	Total (*n* = 57)	Case (*n* = 12)	Control (*n* = 45)	*p*
Gestational age, weeks ^a^	24.6 (23.4, 25.4)	25.3 (24.4, 26.1)	24.6 (23.3, 25)	0.119 ^b^
Birth weight, g ^a^	620 (540, 730)	680 (620, 835)	610 (520, 730)	0.106 ^b^
Male sex	33 (58%)	5 (42%)	28 (62%)	0.200 ^c^
Apgar score ^a^				
At 1 min	4 (3, 5)	4 (2.5, 5.5)	4 (3, 5)	0.584 ^b^
At 5 min	7 (6, 8)	7 (5, 8)	7 (6, 9)	0.301 ^b^
Small for gestational age	13 (23%)	2 (17%)	11 (24%)	0.713 ^c^
Chorioamnionitis	28 (49%)	5 (42%)	23 (51%)	0.561 ^c^
Premature rupture of membrane	17 (30%)	3 (25%)	14 (31%)	1.000 ^c^
Antenatal corticosteroid use	56 (98%)	11 (92%)	45 (100%)	0.211 ^c^
High-grade intraventricular hemorrhage	21 (37%)	3 (25%)	18 (40%)	0.504 ^c^
Neonatal sepsis	52 (91%)	8 (67%)	44 (98%)	0.006 ^c^
Acute kidney injury	22 (39%)	4 (33%)	18 (40%)	0.841 ^c^
NSAIDs use	12 (21%)	4 (33%)	8 (18%)	0.254 ^c^
HFOV support	32 (56%)	5 (42%)	27 (60%)	0.418 ^c^
Preoperative pletelet counts, ×10^9^/L ^d^	154.1 ± 130.0	235.3 ± 161.5	132.4 ± 112.7	0.057 ^b^
Postnatal age at laparotomy, days ^a^	21 (14, 36)	30 (22, 45.5)	20 (12, 31)	0.018 ^b^

Unless otherwise indicated, data are the number of patients, with percentages in parentheses. HFOV, high-frequency oscillatory ventilation. ^a^ Data are the median and interquartile range. ^b^ The *p* values are from a Wilcoxon rank sum test. ^c^ The *p* values are from Fisher’s exact test or a Chi-Square test, as appropriate. ^d^ Data are the mean and standard deviation.

**Table 2:** Comparisons of imaging findings between the two groups.

**Abdominal Radiographs**	**Total (*n* = 57)**	**Case (*n* = 12)**	**Control (*n* = 45)**	***p***
Free air	45 (79%)	0 (0%)	45 (100%)	
Bowel gas pattern				1.000
1. Normal	0	0 (0%)	0 (0%)	
2. Decreased gas or gasless	10 (18%)	2 (17%)	8 (18%)	
3: Ileus without elongated loops	7 (12%)	1 (8%)	6 (13%)	
4. Ileus with elongated loops	40 (70%)	9 (75%)	31 (69%)	
Pneumatosis	19 (33%)	3 (25%)	16 (36%)	0.732
Portal venous gas	1 (2%)	0 (0%)	1 (2%)	1.000
**Bowel US**	**Total (*n* = 48)**	**Case (*n* = 12)**	**Control (*n* = 36)**	***p***
Interval between preoperative US and laparotomy, days	3.5 ± 2.4	2.2 ± 2.6	3.9 ± 2.3	0.029
Free air	9 (19%)	1 (8%)	8 (22%)	0.416
Portal venous gas	3 (6%)	0 (0%)	3 (8%)	0.563
Pneumatosis	21 (44%)	4 (33%)	17 (47%)	0.510
Abdominal fluid	48 (100%)	12 (100%)	36 (100%)	
Complex ascites or focal fluid collections	39 (81%)	12 (100%)	27 (75%)	< 0.001
Simple ascites	2 (4%)	0 (0%)	2 (6%)	1.000
Bowel wall thickening	45 (94%)	12 (100%)	33 (92%)	0.563
Absent or decreased bowel perfusion	7 (15%)	3 (25%)	4 (11%)	0.345
Interval between first detection of complex ascites or focal fluid collections on US and laparotomy, days	6.2 ± 5.6	4.3 ± 4.1	7.1 ± 6.0	0.158

Data are the number of patients, with percentages in parentheses. The *p* values are from Fisher’s exact test or an χ^2^ test, as appropriate.

**Table 3:** Primary and secondary outcomes.

	Case (*n* = 12)	Control (*n* = 45)	*p*	Multivariable Analyses	
				Adjusted OR/Estimate	*p* ^f^
*Primary outcome*					
Death before discharge	1 (8%)	20 (44%)	0.040 ^d^	0.02 (0.00, 0.61) ^a^	0.025
*Secondary outcomes*					
Short bowel syndrome	1 (8%)	5 (11%)	1.000 ^d^	2.25 (0.13, 38.2) ^a^	0.576
TPN dependence for 3 months or more	2 (17%)	7 (16%)	1.000 ^d^	1.79 (0.20, 16.15 ^a^	0.603
Length of hospital stay, days	135 (126, 163) ^b^	133 (121, 209) ^b^	0.797 ^e^	−16.64 (55.92) ^c^	0.770
Bowel stricture requiring surgery	1 (8%)	5 (11%)	1.000 ^d^	0.10 (0.00, 5.49) ^a^	0.262
Sepsis after laparotomy	2 (17%)	19 (42%)	0.177 ^d^	0.31 (0.02, 5.37) ^a^	0.425
Acute kidney injury after laparotomy	1 (8%)	21 (47%)	0.019 ^d^	0.02 (0.00, 1.00) ^a^	0.050
Body weight at 36 weeks PMA, g	1690 (1460, 1970) ^b^	1535 (1230, 1810) ^b^	0.007 ^e^	297.64 (226.58) ^c^	0.210

Unless otherwise indicated, data are the number of patients, with percentages in parentheses. *OR* odds ratio, *TPN* total parenteral nutrition, and *PMA* postmenstrual age. ^a^ Data are adjusted odds ratio with 95% confidence intervals in parentheses. Odds ratio adjusted by gestational age, HFOV support, preoperative platelet counts, and the interval between first detection of complex ascites or focal fluid collection on US and laparotomy. ^b^ Data are the median and interquartile range. ^c^ Data are the estimate with the standard error in parentheses. ^d^ The *p* values are from Fisher’s exact test. ^e^ The *p* values are from a Student’s *t*-test or Wilcoxon rank sum test, as appropriate. ^f^ The *p* values are from logistic or linear analysis, as appropriate.

## Data Availability

Available upon reasonable request.

## Abbreviations

AKI = Acute kidney injury; CI = Confidence interval; IQR = Interquartile range; IVH = Intraventricular hemorrhage; NEC = Necrotizing enterocolitis; NICU = Neonatal intensive care units; OR = Odds ratio; US = Ultrasonography; PMA = Postmenstrual age
